# Relative Contributions of Myostatin and the GH/IGF-1 Axis in Body Composition and Muscle Strength

**DOI:** 10.3389/fphys.2018.01418

**Published:** 2018-11-01

**Authors:** Nicholas R. Lozier, John J. Kopchick, Sonsoles de Lacalle

**Affiliations:** Department of Biomedical Sciences, Heritage College of Osteopathic Medicine, Ohio University, Athens, OH, United States

**Keywords:** myostatin, growth hormone, sarcopenia, body composition, IGF-1, grip strength, muscle hypertrophy

## Abstract

Myostatin, a negative regulator of muscle growth, is considered a potential therapeutic agent for individuals suffering from various muscle wasting and strength declining diseases because inhibiting Mstn signaling leads to muscular hypertrophy. In this study we investigate the interaction between myostatin and the growth hormone/insulin-like growth factor-1 (GH/IGF-1) axis in muscle function and strength. To this end, we measured hind limb grip strength and myostatin levels in two mouse models of GH gene manipulation; GH receptor knockout (*GHR^-/-^)* mice which have reduced GH/IGF-1 action, and bovine GH transgenic (bGH) mice which have excess GH/IGF-1 action. We found that specific muscle force was significantly reduced in bGH mice, and significantly increased in *GHR^-/-^* mice, compared to their respective littermate wild type controls. The expression of the mature form of myostatin was significantly increased in bGH mice, and unchanged in *GHR^-/-^* mice. In the bGH mice, the high levels of mature myostatin were accompanied by increase body weight and lean mass, consistent with other published results indicating that the IGF-1 signaling pathway is dominant over that of Mstn. Our results also suggest that in these mouse models there is an inverse relationship between muscle strength and levels of myostatin and GH, since constitutive overexpression of GH resulted in elevated levels of mature myostatin in muscle, accompanied by a reduction in strength. By contrast, in the *GHR^-/-^* mice with reduced levels of IGF-1, mature myostatin levels were unchanged and muscle strength was increased.

## Introduction

Myostatin (Mstn), a member of the transforming growth factor beta (TGF-β) cytokine superfamily, is a negative regulator of muscle growth (McPherron et al., 1997) known to inhibit muscle satellite cell differentiation during early development (McCroskery, 2003; Artaza et al., 2005) and in adulthood (Welle et al., 2007). Mstn is synthesized as a 376 amino acid protein, dimerizes post translation and undergoes complex proteolytic treatment resulting in the mature, ligand form of Mstn (∼26 kDa) (Thomas et al., 2000; Wolfman et al., 2003; Lee, 2004; Artaza et al., 2005; Huang et al., 2011).

Muscle wasting is linked to excess Mstn in circulation (Gonzalez-Cadavid et al., 1998; Marcell et al., 2001; Costelli et al., 2008). Mice overexpressing Mstn have less skeletal muscle than littermate controls (Thomas et al., 2000; Lee, 2004; Huang et al., 2011), while *MSTN* gene mutations and reduced protein levels increase muscle mass (Kambadur et al., 1997; Lin et al., 2002; Schuelke et al., 2004; Mosher et al., 2007; Williams et al., 2015).

Counter to Mstn action, activation of the growth hormone/insulin-like growth factor-1 (GH/IGF-1) axis results in muscle growth, and attempts at clarifying these opposing actions have offered somewhat inconsistent results (Lalani et al., 2000; Marcell et al., 2001; Liu et al., 2003; Oldham et al., 2009; Brooks et al., 2011; Price et al., 2011). While both GH and IGF-1 are associated with increased muscle performance (Gläser et al., 2010; Taekema et al., 2011), the effect of Mstn on muscle strength remains unclear. We have reported (Williams et al., 2015) that *MSTN* inhibition may enhance overall strength due to hypertrophy, but that specific muscular force is not significantly different when normalized to muscle wet weights. Other studies have found that muscle fiber function is significantly reduced in *MSTN^-/-^* mice relative to muscle weights (Amthor et al., 2007; Gentry et al., 2011).

Here we report the results of investigating Mstn levels and muscle strength in two mouse models of GH/IGF-1 dysfunction referred to as the bovine GH transgenic mouse (bGH) and the GH receptor/GH binding protein knockout mouse (*GHR^-/-^)*, both of which previously characterized (Zhou et al., 1997; Berryman et al., 2004; Palmer et al., 2009; List et al., 2011). In *GHR^-/-^* mice there is a disruption of the genes for GHR and GH binding protein; these mice are dwarf, obese, insulin sensitive, GH resistant, have greatly reduced IGF-1 and elevated GH serum levels. In bGH mice, the insertion of a transgenic bovine GH gene leads to GH overexpression, and these mice have increased body size, higher percent lean mass, lower percent body fat, and higher circulating IGF-1 and insulin than controls.

## Materials and Methods

Experimental subjects were 7-month-old male genetically modified mice, and their wild-type littermates served as controls, all previously characterized in detail (Zhou et al., 1997; Berryman et al., 2004; List et al., 2011). Protocols were approved by Ohio University’s Institutional Animal Care and Use Committee. Mice were fed *ad libitum* a standard lab chow diet and kept on a 14/10 h light/dark cycle.

Body composition data were collected using a Bruker Minispec (The Woodlands, TX, United States) as described previously (Berryman et al., 2004). To test for grip strength mice (*GHR^-/-^ n* = 9, controls *n* = 7 and bGH *n* = 7, controls *n* = 5) were scruffed and held above a computerized mesh grid (San Diego Instruments), as described by Hakim et al. (2011). Scores were recorded in grams of force in sets of 5, with the average coming from the 3 highest scores per individual. Given that a recent recommendation to use absolute grip strength values (Takeshita et al., 2017) has been validated for aging studies, for our young subjects we chose to use raw scores normalized to body weight, as employed by most in the field (for example, Aono et al., 2011; Hakim et al., 2011), which would allow for better comparisons across studies.

Triceps surae muscles were harvested from an additional group of bGH mice (*n* = 10), *GHR^-/-^* mice (*n* = 8), and equivalent numbers of controls for each, flash frozen in liquid nitrogen, and stored at -80^°^C until use. Samples were then subject to subcellular fractionation as described (Cox and Emili, 2006; Dimauro et al., 2012) to obtain the cytosolic fraction, predicted to contain only precursor Mstn prior to its secretion; a reflection of the amount of Mstn inside the muscle cell.

**FIGURE 1:**
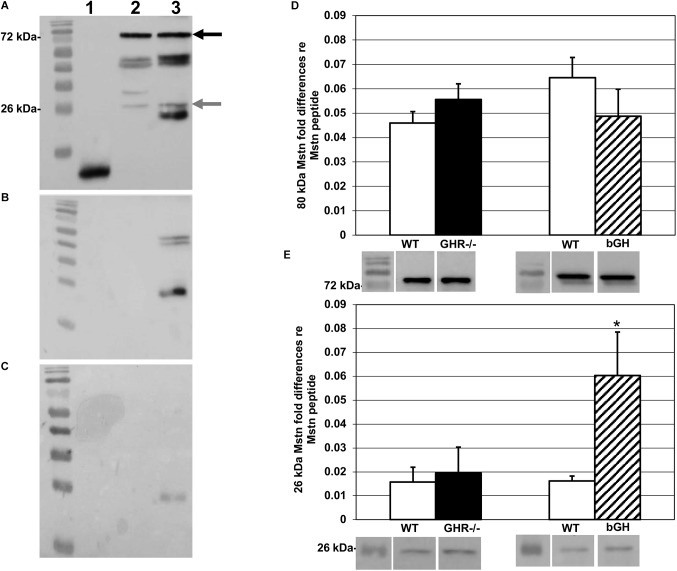
Myostatin protein levels. Western blots probed with the anti-Mstn monoclonal antibody described in the text. In blots **A–C**, lane 1 contains synthetic Mstn peptide; lane 2, rat triceps surae homogenate; lane 3, mouse triceps surae homogenate. **(A)** Blot probed with primary and secondary antibody (see text for details). Precursor protein band is indicated by black arrow (80 kDa) and mature protein by gray arrow (26 kDa). **(B)** Blots probed with secondary antibody alone to test specificity showed non-specific binding in mouse triceps surae homogenate (lane 3). For synthetic Mstn and rat triceps surae (lanes 1 and 2, respectively), we did not find non-specific binding. **(C)** To reduce non-specific binding, non-conjugated anti-Fab antibody was added to blocking buffer prior to the addition of primary antibody, and this procedure was applied then to all samples. **(D)** Quantification of levels of precursor Mstn protein, relative to the synthetic Mstn loading control, expressed as fold-change ±SEM. Compared to controls, there was no change in levels of precursor Mstn protein in either group. Representative Western blots are shown under the respective bars. **(E)** Quantification of levels of mature Mstn protein, relative to the synthetic Mstn loading control, expressed as fold-change ±SEM. There was no change in levels of mature Mstn protein in *GHR^-/-^* mice (*n* = 8) compared to controls. bGH mice (*n* = 10) expressed significantly more mature Mstn protein compared to controls, ^∗^*p* < 0.05. Representative Western blots are shown under the respective bars.

Western blots of the cytosolic fraction were resolved in triplicate with 0.8 ng of Mstn peptide as a transfer control per gel. Each sample was diluted with Laemmli buffer to 25 μl and loaded on 40%, 37.5:1 acrylamide/bis-acrylamide (4% stacking and 12% resolving) gels, electrophoresed for 70 min at 35 mAmps/gel, and transferred onto nitrocellulose membranes at 100 V for 60 min. Membranes were blocked with 5% milk/TBS-Triton X (TBS-T) and probed with a monoclonal (1:5000) anti-Mstn primary antibody from Oxford Brookes University (Oxford, United Kingdom). This antibody is specific to a 113 amino acid sequence on the C-terminal region of human Mstn, and 100% conserved between mouse, rat, and human (Artaza et al., 2005). Control Western blots using Mstn synthetic peptide (R&D Systems, MN, United States), rat triceps surae, mouse triceps surae, and mouse soleus showed bands at both 80 and 26 kDa, confirming that bands seen in mouse muscle were conserved forms of precursor and mature Mstn, respectively (Figure 1A).

The secondary antibody was an HRP-conjugated rabbit anti-mouse Immunoglobulin G (IgG) (Cell Signaling Technology, MA, United States; 1:3000 in 5% milk/TBS-T). Control blots incubated without primary antibody showed non-specific binding of the secondary antibody (Figure 1B), so we added antigen-binding fragment (Fab) anti-IgG blocking antibody (Jackson ImmunoResearch, PA, United States; 1:500), which effectively reduced non-specific binding (Figure 1C). Blots were imaged with the ChemiDoc XRS+ system (Bio-Rad, CA, United States) and band volumes (intensity × area, arbitrary units) quantified (Figures 1D,E) using Image Lab software (Bio-Rad, CA, United States); fold differences were calculated as described (Taylor and Posch, 2014).

A value of *p* < 0.05 indicated significance in all statistical analyses. Body weight and percent lean mass were analyzed in independent samples *t*-tests, two-tailed, comparing each mutant mouse line with its respective control. *GHR^-/-^* and bGH mice were not directly compared because they were not littermates, and although both mutant strains originated from the same genetic background (C57/BL6N), the two strains had been backcrossed for multiple generations but not using the same C57/BL6N mice. In Western blots, band volume fold differences were analyzed in an independent samples *t*-test, two-tailed. A few bands were non-quantifiable due to bubbles on membrane hence slight discrepancies in the degrees of freedom for the *t*-tests. For hind limb grip strength assessment, the mean of the three highest grip strength scores (out of 5) per individual were averaged and analyzed in an independent samples *t*-test, two-tailed.

## Results

We had originally predicted finding solely precursor Mstn due to earlier reports describing extracellular proteolytic processing of mature Mstn (Thomas et al., 2000; Lee, 2004; Artaza et al., 2005; Huang et al., 2011). However, Western blotting of the cytosolic fraction extracted from the muscle samples revealed the presence of precursor and mature forms of Mstn, with bands at 80 and 26 kDa, respectively. Both bands were quantified in our samples. The levels of expression of precursor and mature Mstn in *GHR^-/-^* mice (*M* = 0.056 ± 0.006 and *M* = 0.020 ± 0.011 respectively) were not different from littermate controls (*M* = 0.046 ± 0.005 and *M* = 0.016 ± 0.006 respectively) [*t*(47) = -1.24, *p* = 0.22 and *t*(43) = ±0.31, *p* = 0.76; Figures 1D,E], whereas bGH mice had significantly higher levels of mature (*M* = 0.06 ± 0.018) [*t*(42) = ±2.92, *p* < 0.05] but not of precursor (*M* = 0.049 ± 0.011) Mstn [*t*(36) = ±1.16, *p* = 0.25], compared to controls (*M* = 0.0016 ± 0.002 and *M* = 0.065 ± 0.008 respectively) (Figures 1D,E).

**FIGURE 2:**
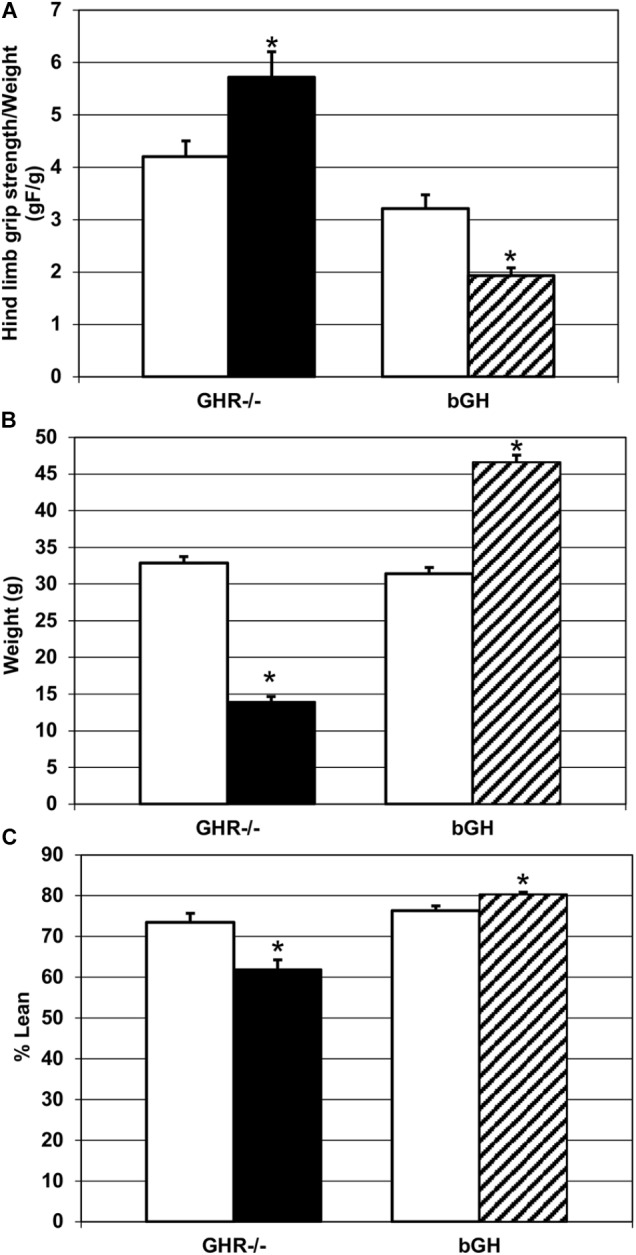
Changes in strength and phenotype. **(A)** Quantification of hind limb grip strength in each experimental group. *GHR^-/-^* mice (*n* = 9) were significantly stronger than controls (*n* = 7), and bGH mice (*n* = 7) were significantly weaker than controls (*n* = 5), ^∗^*p* < 0.05. **(B)** Quantification of body weight change in each experimental group. *GHR^-/-^* mice (*n* = 9) weighed significantly less than controls (*n* = 7) and bGH mice (*n* = 7) weighed significantly more than controls (*n* = 5), ^∗^*p* < 0.05. **(C)** Quantification of percent lean mass change in each experimental group. *GHR^-/-^* mice (*n* = 9) had significantly reduced percent lean mass, compared to controls (*n* = 7), and bGH mice (*n* = 7) had significantly increased percent lean mass, compared to controls (*n* = 5), ^∗^*p* < 0.05.

Comparison of hind limb grip strength (Figure 2A) revealed that *GHR^-/-^* mice were significantly stronger (*M* = 5.72 ± 0.48) than controls (*M* = 4.21 ± 0.3), as determined by higher specific muscle force [*t*(14) = ±2.47, *p* < 0.05]. In contrast, bGH mice exhibited significantly lower specific muscle force (*M* = 1.91 ± 0.15) than their controls (*M* = 3.21 ± 0.26) [*t*(10) = ±4.59, *p* < 0.05]. Those results were unexpected given that *GHR^-/-^* mice weighed significantly less (*M* = 13.95 ± 0.72) [*t*(16) = ±16.4, *p* < 0.05] and contained significantly less percent lean mass (*M* = 61.98 ± 2.29) than controls (*M* = 32.89 ± 0.86 and *M* = 73.43 ± 2.26 respectively) [*t*(16) = ±3.52, *p* < 0.05], Figures 2B,C. In agreement with other studies (List et al., 2011), bGH mice were significantly heavier (*M* = 46.61 ± 0.97) than controls (*M* = 31.45 ± 0.81) [*t*(18) = ±11.98, *p* < 0.05] and had higher % lean mass (*M* = 80.34 ± 0.53) than controls (*M* = 76.29 ± 1.22) [*t*(18) = ±3.04, *p* < 0.05], Figures 2B,C.

## Discussion

This short communication reports cytosolic levels of Mstn in two mice models in which body composition is drastically altered. Elucidating the relative role that Mstn and the GH/IGF-1 axis play in muscle function is extremely important to find a treatment for dynapenia and muscle wasting diseases. *GHR^-/-^* and bGH mice provide a functional and unique model in which to address this question by determining how dysregulation of GH action impacts muscle function and whether this impact could be related to Mstn levels. Muscle strength in these animal models has not been previously published.

The interaction of Mstn and IGF-1 signaling pathways within myocytes has been described to some degree. A cell culture study by Trendelenburg et al. (2009) reported that myostatin inhibits activation of Akt, in both myoblasts and myotubes, an effect blocked by the addition of IGF-1, suggesting that IGF-1/Akt signaling is dominant over myostatin-induced inhibition of Akt. Interestingly, IGF-1 mediates physiological hypertrophy of heart muscle, but an excess of IGF-1 can lead to cardiac dysfunction, as shown in acromegaly, a disease characterized by excessive production of GH which stimulates massive release of IGF-1 (Sacca et al., 1994). Shyu et al. (2005) have shown that IGF-1 in cultured rat neonatal cardiomyocytes stimulates p38 MAPK to induce expression of Mstn and compensate excessive growth. In agreement with this model, our bGH mice also exhibited higher levels of Mstn, suggesting that the mechanism proposed for heart could also be applied to skeletal muscle. Previous work (Consitt et al., 2017) has shown that levels of p38 MAPK are significantly elevated in bGH mice compared to controls, as well as levels of Mstn. Our results confirm and refine some of these findings. While we also found increased levels of the active peptide, we did not see changes in the levels of precursor Mstn. This could be due to experimental differences, since we chose to process the whole triceps surae muscle to avoid potential sources of error in the dissection of each component, and we also applied a subcellular fractionation technique that allows for a more accurate measurement of the cytosolic content. Since there were no changes in the cytosolic levels of precursor Mstn, one possible explanation for increased mature Mstn in bGH muscle is that over-secretion of IGF-1 leads to increased proteolytic production of mature Mstn protein from myocytes. In this regard it will be interesting to explore whether p38 MAPK is the link between Mstn and IGF-1 intracellular cascades in skeletal muscle. From a technical standpoint, it could be argued that higher levels of mature Mstn in the bGH samples could be the product of proteolysis as the tissue thawed, or the result of artificial activation induced by heat when preparing samples for blots (Wolfman et al., 2003). This is unlikely because the change was only seen in one group of mice, whereas all tissues were prepared at the same time following identical procedure.

We also investigated whether changes in body composition in these two animal models had an impact on muscle strength. Our results suggest that the increase in IGF-1 accompanied by higher levels of Mstn active peptide impair muscle strength despite increase in body weight and lean mass, as shown in the bGH mice. Even with the decrease in body weight and lean mass, *GHR^-/-^* mice were stronger than littermate controls.

These findings suggest that balance between IGF-1 and Mstn, and not body or muscle mass, is more important for the maintenance of muscle strength. Future work investigating neuromuscular physiology in these mutant mouse models will provide important information as to the mechanisms of the GH/IGF-1 axis and muscular performance.

While the differences in body composition exhibited by the adult *GHR^-/-^* and bGH mice do not appear mediated by Mstn levels, since these are constitutive genetic models, it will be also important to identify potential developmental effects of the genetic modifications, before concluding on possible signaling cascades that may be useful in the design of treatments for muscle wasting. We speculate that instead of only increasing muscle mass, future therapeutic approaches may investigate whether reducing IGF-1 while maintaining Mstn levels in adults, might be able to ameliorate muscle strength.

## Author Contributions

NL, JK, and SdL contributed to conception, design, and execution of the study. NL and SdL wrote the first draft of the manuscript. All authors contributed to manuscript revision, read, and approved the submitted version.

## Conflict of Interest Statement

The authors declare that the research was conducted in the absence of any commercial or financial relationships that could be construed as a potential conflict of interest.
